# A *Brucella* Omp16 Conditional Deletion Strain Is Attenuated in BALB/c Mice

**DOI:** 10.4014/jmb.2107.07016

**Published:** 2021-10-20

**Authors:** Feijie Zhi, Jiaoyang Fang, Weifang Zheng, Junmei Li, Guangdong Zhang, Dong Zhou, Yaping Jin, Aihua Wang

**Affiliations:** 1 College of Veterinary Medicine, Northwest A&F University, Yangling 712100, P.R. China; 2 Key Laboratory of Animal Biotechnology of the Ministry of Agriculture, Northwest A&F University, Yangling 712100, P.R. China

**Keywords:** *Brucella suis* S2, Omp16, virulence, immune response, mice

## Abstract

*Brucella* spp. are facultative intracellular pathogens that invade, survive and proliferate in numerous phagocytic and non-phagocytic cell types, thereby leading to human and animal brucellosis. Outer membrane proteins (Omps) are major immunogenic and protective antigens that are implicated in *Brucella virulence*. A strain deleted of the *omp16* gene has not been obtained which suggests that the Omp16 protein is vital for *Brucella* survival. Nevertheless, we previously constructed an *omp16* conditional deletion strain of *Brucella*, ΔOmp16. Here, the virulence and immune response elicted by this strain were assessed in a mouse model of infection. Splenomegaly was significantly reduced at two weeks post-infection in ΔOmp16-infected mice compared to infection with the parental strain. The bacterial load in the spleen also was significantly decreased at this post-infection time point in ΔOmp16-infected mice. Histopathological changes in the spleen were observed via hematoxylineosin staining and microscopic examination which showed that infection with the ΔOmp16 strain alleviated spleen histopathological alterations compared to mice infected with the parental strain. Moreover, the levels of humoral and cellular immunity were similar in both ΔOmp16-infected mice and parental strain-infected mice. The results overall show that the virulence of ΔOmp16 is attenuated markedly, but that the immune responses mediated by the deletion and parental strains in mice are indistinguishable. The data provide important insights that illuminate the pathogenic strategies adopted by *Brucella*.

## Introduction

Brucellosis is an endemic zoonotic bacterial disease caused by *Brucella* spp. which are able to establish persistent infection in hosts [1, 2]. The *Brucella* genus currently comprises six classical species based on natural host specificity [3], although novel *Brucella* species continue to be discovered, including *Brucella ceti*, *B. inopinata*, *B. microti*, and *B. pinnipedialis* [4, 5]. Among the most well-characterized species, *B. abortus*, *B. canis*, *B. melitensis*, and *B. suis* infect humans with most cases attributed to *B. melitensis* [6, 7]. The main symptoms of *Brucella* infection in non-human animals are spontaneous abortion at late gestation, infertility and orchitis which lead to devastating economic and social losses [8-10]. In contrast, brucellosis in humans is characterized by a long incubation period that leads to a variety of atypical clinical symptoms, including undulant fever, osteoarticular pain, splenomegaly, and hepatomegaly. More than 500,000 human cases of *Brucella* infection are reported annually worldwide [7]. *Brucella* vaccines that currently are adminstered in animals have certain disadvantages, including sero-diagnostic interference and residual pathogenicity [11-13]. No approved *Brucella* vaccines are available for human use [9].

One of the main characteristics of *Brucella* spp. is the absence of certain classic virulence factors, including cytolysins, fimbria, exotoxins, capsules, lysogenic phages, and plasmids, compared to other pathogenic bacteria [14]. Instead, the main virulence factors in *Brucella* include type IV secretion systems (T4SS), lipopolysaccharides (LPS), the BvrR/BvrS two-component system, cyclic β-1-2-glucans (CβG), and outer membrane proteins (Omps) that mediate interaction with the host cell surface or related signaling pathways to promote bacterial survival [7, 15, 16]. A functional T4SS system is required for *Brucella* survival in vivo and in vitro, and T4SS effectors, such as VceC, BspA, BspB, and BspB, contribute to *Brucella* intracellular proliferation [17, 18]. LPS in *Brucella* exhibits properties that differ from LPS in other gram-negative bacteria. For example, LPS of *Brucella* is less toxic and less active than *Escherichia coli* LPS [16]. *Brucella* CβG is required for survival in HeLa cells and proliferation in mice [19]. In addition, other factors involved in *Brucella virulence* and that play different roles in pathogenicity include urease, cytochrome oxidase, nitric oxide reductase, and *Brucella virulence* factor A (BvfA) [15, 16].

Omps in *Brucella* are exposed at the bacterial surface and are implicated strongly in virulence [20, 21]. For example, the Omp10 deletion strain is defective for growth in minimal medium and infection is attenuated in mice [22, 23]. Deletion of Omp19 reduces intracellular survival in macrophages and also attenuates murine infection [24, 25]. Omp25 and Omp31 also contribute to intracellular survival of *Brucella* in vitro and in vivo, as well as to chronic infection [20, 26]. Omp16 is a pathogen-associated molecular pattern (PAMP) that is highly conserved in all *Brucella* species and which activates dendritic cells (DCs) and which also induces a T helper 1 (Th1) immune response [27]. In addition, Omp16 can alter *Brucella*-mediated immune-related pathways in macrophages. However, *Brucella* deleted of the *omp16* gene has not been acquired, which suggests that the Omp16 protein is vital for survival in this bacterium [28]. Nevertheless, we previously generated a derivative of *B. suis* strain 2 (*B. suis* S2) in which *omp16* expression was controlled (ΔOmp16). However, the virulence and immune response mediated by the ΔOmp16 strain have not been evaluated in mice.

In this study, our goal was to examine the effect of Omp16 on the virulence and immunity of *Brucella* in BALB/c mice by analyzing splenomegaly, spleen bacterial load, spleen histopathology, and humoral and cellular immunity. We show that Omp16 is required for *Brucella* survival in vivo and that the levels of humoral and cellular immunity in ΔOmp16-infected mice were similar to those in wild-type strain-infected mice. These findings indicate that Omp16 is important for the virulence of *Brucella*, but that the immune response is not altered by the absence of the protein. The study provides new insights into the crucial role of Omp16 in *Brucella virulence* and into a broader understanding of the mechanisms of *Brucella* pathogenicity.

## Materials and Methods

### Bacterial Strains and Culture Conditions

Wild-type *B. suis* S2 (CVCC reference number CVCC70502) and ΔOmp16 have been described previously [29]. *B. suis* S2 and ΔOmp16 were grown on tryptic soy agar (TSA; Sigma, USA) for 72 h at 37°C and 5% CO_2_ and then cultured in tryptic soy broth (TSB; Sigma) at 37°C with shaking to an optical density at 600 nm (OD_600_) of ~0.6. For infection, *B. suis* S2 and ΔOmp16 in the logarithmic growth phase were collected by centrifugation and the numbers of bacteria were determined using 10-fold gradient dilutions.

### Mice Infection

Groups of six-to-eight-week old BALB/c female mice (Experimental Animal Center, Xi'an Jiaotong University, Shaanxi, China) were acclimated at least one week prior to infection. Each experimental group contained at least five mice. The mice were inoculated intraperitoneally with 10^7^ colony-forming units (CFU) of *B. suis* S2 or with the ΔOmp16 strain in 200 μl of phosphate-buffered saline (PBS). The infected mice were monitored daily for survival. The mice were sacrificed by cervical dislocation at one, two and four weeks post-infection. The spleens from infected mice were removed and weighed to evaluate splenomegaly. To determine the bacterial load, one-third of each spleen was homogenized in 0.5 ml of PBS. Tissue homogenates were diluted by a 10-fold gradient and spread on TSA plates. CFU were counted after 72 h of incubation at 37°C.

### Histological and Immunohistological Analysis

Spleen tissues from infected mice were collected at one, two and four weeks post-infection. One-third of each spleen was fixed in 4% paraformaldehyde for three days at room temperature. After fixation, the tissues were embedded in paraffin blocks which were then sectioned into 5 μm slices using a microtome. The sections were transferred to glass slides and allowed to adhere prior to further processing. The sections were examined routinely with hematoxylin-eosin staining (H&E staining) to evaluate pathological features. Immunohistochemical staining was performed to observe the bacterial load in spleen samples. Then, the sections were then treated with 3% H_2_O_2_ for 30 min at room temperature to block endogenous peroxidase activity. Next, the sections were blocked in PBS with 1% BSA for 1 h at room temperature before being permeabilized in PBS containing 0.25% Triton X-100 at room temperature for 30 min. Finally, the sections were incubated with primary anti-brucella goat polyclonal antibody (1:100 dilution) overnight at 4°C. Donkey anti-goat Alexa Fluor 555 (Invitrogen, USA; 1:500 dilution) was used as the secondary antibody. Subsequently, slides were observed under a laser scanning confocal microscope (Nikon).

### RNA Isolation and Quantitative Real-Time PCR

Spleen tissues from infected mice were collected at one, two and four weeks post-infection. One-third of each spleen was homogenized in 0.5 ml of TRIzol (Invitrogen). Total RNA was extracted from the spleen tissues using TRIzol according to the manufacturer's protocol. Reverse transcription was performed using the RevertAid First Strand cDNA Synthesis Kit (Thermo Fisher Scientific, USA) according to the manufacturer’s recommended protocols. SYBR Premix Ex Taq (Vazyme, China) and the ABI 7500 Sequencing Detection System (Applied Biosystems, USA) were used for quantitative real-time PCR (qRT-PCR). The relative transcription levels were analyzed by the 2^-ΔΔCt^ method [30]. All the primers used are listed in Table 1.

### Detection of Cytokine Production

The serum from infected mice was separated by centrifugation at 1,000 ×g at 4°C for 10 min and then stored at -80°C until analysis. Serum IFN-γ, IgG, IgG1, and IgG2a were detected by mouse IFN-γ, IgG, IgG1, and IgG2a ELISA kits, respectively, according to the manufacturer’s (Multi Sciences [Lianke], China) instructions.

### Statistical Analysis

Statistical analysis was performed using SPSS version 23 software. All results are presented as standard deviations (SD) and were repeated at least three times. Statistical significance was performed using unpaired, two-tailed Student’s *t*-test or two-way analysis of variance (ANOVA) followed by either Bonferroni’s or Sidak’s multiple-comparison test. Probability (*p*) < 0.05 was considered statistically significant. *p* ≤ 0.01 are denoted by **; 0.01 < *p* < 0.05 are denoted by *.

### Ethics Statement

All animal experiments were conducted in accordance with the “Guidelines on Ethical Treatment of Experimental Animals” (2006) No. 398 from the Ministry of Science and Technology, China. The sampling procedures used in the study received prior approval from the Experimental Animal Management Committee of Northwest A&F University with the approval license number 2018ZX08018023.

## Results

### Analysis of Spleen Morphology

**Fig. 1:**
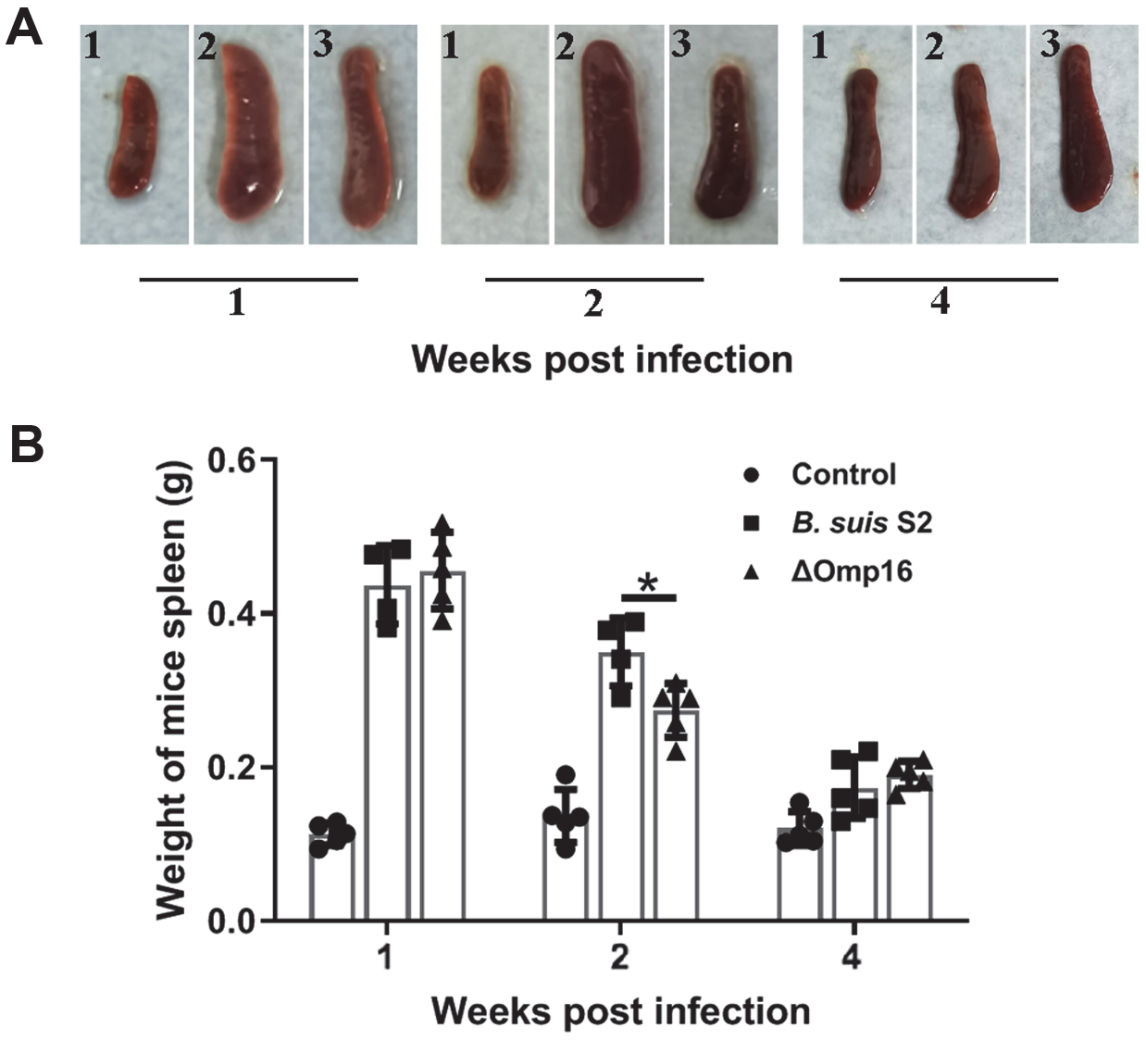
Splenomegaly and spleen weights of mice infected with *B. suis* 2 wild-type or the ΔOmp16 strain. (**A**) Splenomegaly was observed at one, two and four weeks post-infection. 1, spleen of PBS-injected mice; 2, spleen of wild-type strain-infected mice; 3, spleen of ΔOmp16-infected mice. The image shown is representative of at least five independent experiments. (**B**) Spleen weights were measured at one, two and four weeks post-infection. The results are expressed as the means ± standard deviations (SD).

The spleen is the main target organ for colonization and is also the crucial marker of residual bacterial virulence in *Brucella*-infected mice. To determine the effect of the Omp16 protein on bacterial virulence in vivo, six-to-eight-week old female BALB/c mice were injected with approximately 1 × 10^7^ CFU of *B. suis* S2 or the ΔOmp16 strain. The spleens of mice were enlarged significantly in both wild-type strain-infected mice and ΔOmp16-infected mice compared to uninfected mice at one week post-infection, but splenomegaly was not significantly different between mice infected with either wild-type strain or ΔOmp16 (Fig. 1A). The spleens of mice still were enlarged significantly following either wild-type strain or ΔOmp16 infection compared to uninfected mice after two weeks, but splenomegaly at this time point was reduced in ΔOmp16-infected mice compared to mice infected with the parental strain (Fig. 1A). The spleens of both wild-type strain- and ΔOmp16-infected mice were fully restored at four weeks post-infection (Fig. 1A). Consistently, spleen weights were increased in both wild-type strain- and ΔOmp16-infected mice at one and two weeks post-infection, but were fully restored four weeks after infection (Fig. 1B). However, spleen weight was reduced following ΔOmp16 infection compared to wild-type strain infection at the two-week post-infection time point (Fig. 1B). These results demonstrate that Omp16 is involved in *Brucella virulence* in vivo.

### Omp16 Deficiency Impairs *Brucella* Survival in the Mouse Spleen

**Fig. 2:**
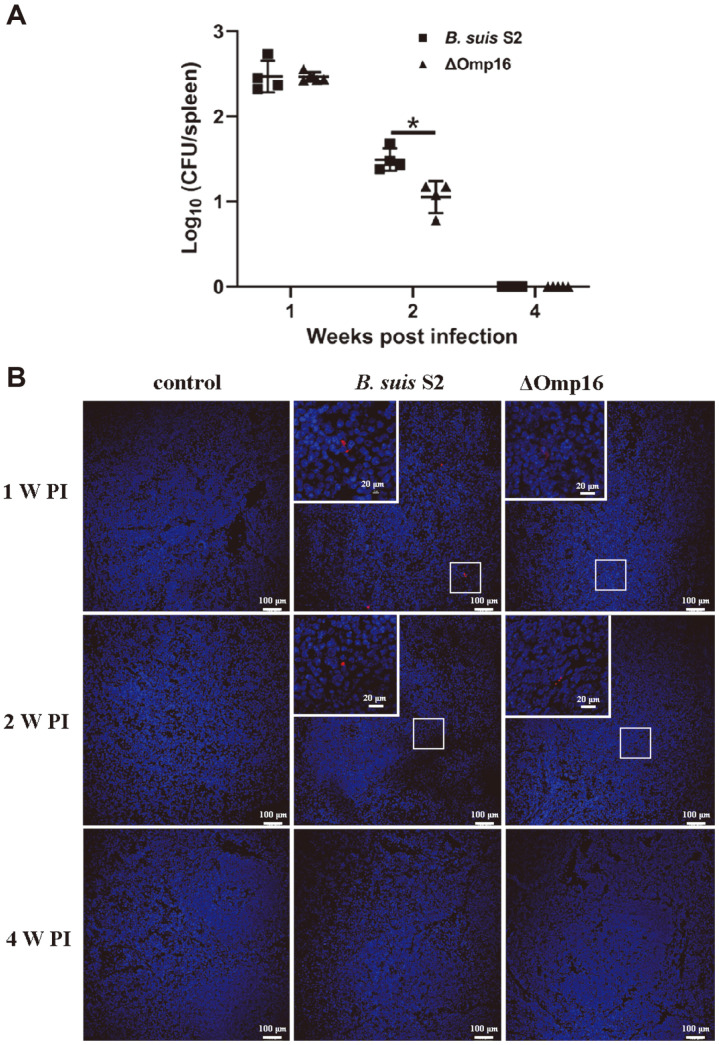
Bacterial survival in the spleens of infected mice. (**A**) The number of bacteria was measured via homogenate at one, two and four weeks post-infection. The results are expressed as the means ± SD. (**B**) Confocal microscopy analysis for bacterial survival in spleen of infected mice at one, two and four weeks post-infection. Red indicates wild-type strain or ΔOmp16 whereas the cell nucleus stains blue. The image shown is representative of at least three independent experiments.

Virulent strains of *Brucella*, including *B. abortus* strain 2308 and *B. melitensis* strain 16M, persist in the mouse spleen and cause long-term infection. However, live attenuated *Brucella* strains, such as *B. abortus* strains RB51 and S19, *B. melitensis* strain Rev1, and *B. suis* strain S2, are eradicated quickly [11, 31, 32]. Based on spleen morphology, we hypothesized that Omp16 might affect *Brucella* survival in the mouse spleen. To this end, the survival of the ΔOmp16 strain in the spleen was examined by determining the CFU number. The numbers of bacteria decreased from one week post-infection to four weeks post-infection in the spleen of both wild-type strain- and ΔOmp16-infected mice (Fig. 2A). The bacterial loads were not significantly different between the wild-type strain and ΔOmp16 strains one week after infection, but the ΔOmp16 strain survived less well two weeks post-infection compared to the parental strain (Fig. 2A). The bacteria were completely eradicated from the spleen in both cases four weeks after infection (Fig. 2A). These results were confirmed by confocal microscopy analysis (Fig. 2B). The results overall indicate that Omp16 is required for efficient survival of *B. suis* in the mouse spleen.

### Pathological Analysis of Mice Immunized with ΔOmp16

**Fig. 3:**
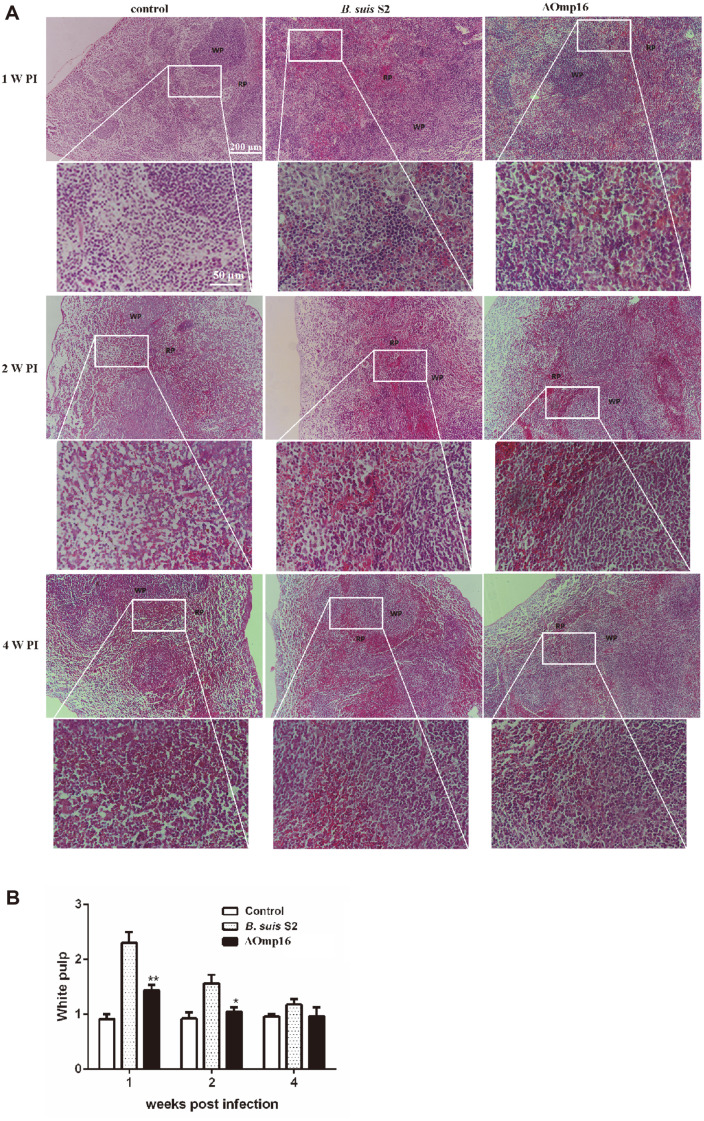
Histopathology of the spleen at one, two and four weeks post-infection with wild-type strain or ΔOmp16. Spleen tissues from infected mice were collected at one, two and four weeks post-infection. After fixation, the tissues were embedded in paraffin blocks which were then sectioned into 5 μm slices using a microtome. The sections were transferred to glass slides and allowed to adhere prior to further processing. The pathological features were assessed via H&E staining. Arrows represent macrophages. WP, White pulp; RP, red pulp.

In view of the preceding characterization of the ΔOmp16 strain in the spleen, histopathologic examination was performed to investigate further the effect of the Omp16<Omp16> deletion on infectivity in mice. To this end, spleen tissues from mice infected either with the S2 strain or ΔOmp16 strain were collected at intervals of one, two, and four weeks post-infection and were fixed, mounted, and subjected to H&E staining to assess tissue sample structure. No significant pathological changes were observed in uninfected spleens at the three time points (Fig. 3). However, spleens infected with the parental strain exhibited a significant increase in the white-to-red pulp ratio due to white pulp expansion at one and two weeks post-infection, although this effect was fully restored four weeks after infection (Fig. 3). In parallel, macrophage numbers increased in the red pulp of spleens of wild-type strain-infected mice after one week compared to uninfected mouse spleens (Fig. 3). In contrast, infection with the ΔOmp16 strain reduced the pathological characteristics of the spleen compared to infection with wild-type strain with a modest increase in the white-to-red pulp ratio and slight white pulp expansion at one week post-infection (Fig. 3). In parallel, macrophage numbers increased in the red pulp of ΔOmp16-infected spleens at one week post-infection (Fig. 3). The data show that the pathological characteristics elicited by wild-type strain were ameliorated in spleens infected with the ΔOmp16 strain.

### Humoral and Cellular Immune Responses Elicited by Vaccination

**Fig. 4:**
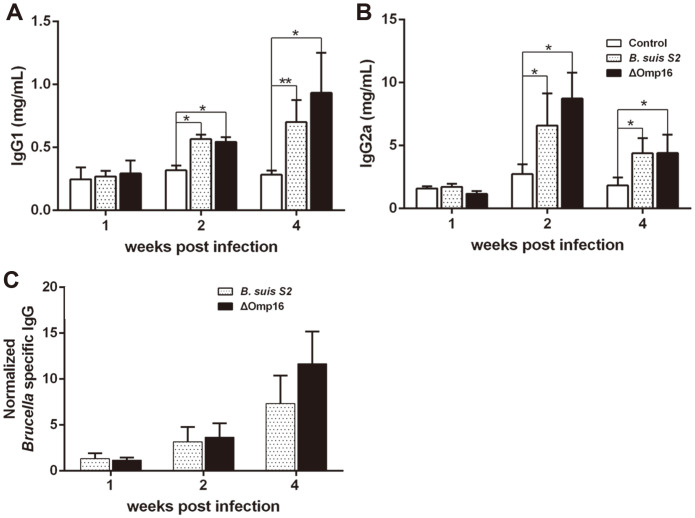
Humoral immune response elicited in mice infected with wild-type strain or ΔOmp16. The serum from infected mice was collected and separated by centrifugation at 1,000 ×g at 4°C for 10 min. IgG1 (**A**) and IgG2a (**B**) were measured at one, two and four weeks post-infection with ELISA kits. The results are expressed as the means ± SD from three independent experiments.

**Fig. 5:**
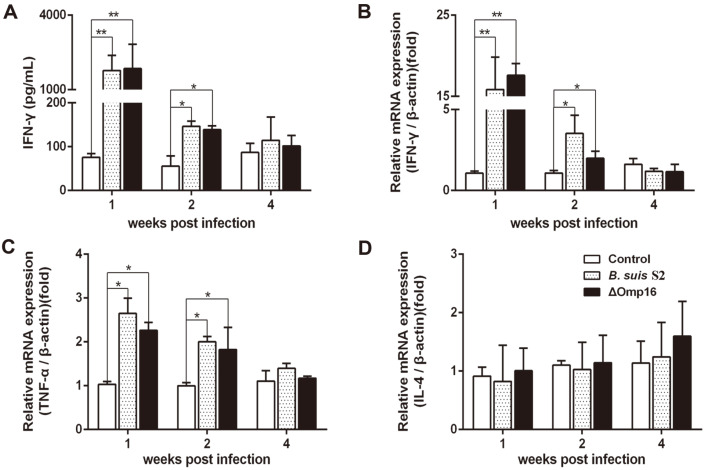
Cytokine expression in serum and splenocytes in mice infected with wild-type strain or ΔOmp16. (**A**) The secretion of IFN-γ in serum of mice was assayed with ELISA kits at one, two and four weeks post-infection. The results are expressed as the means ± SD from three independent experiments. The mRNA levels of IFN-γ (**B**), TNF-α (**C**) and IL-4 (**D**) were assayed by qRT-PCR at one, two and four weeks post-infection. The results are expressed as the means ± SD from three independent experiments.

To further assess the effect of Omp16 on the humoral and cellular immune responses, the spleens from infected mice were collected and IgG1 and IgG2 secretion was detected by ELISA and inflammatory cytokine levels were assessed by qRT-PCR. The secretion of IgG1 and IgG2 from serum was increased in wild-type strain-infected mice at two and four weeks post-infection compared to uninfected mice (Fig. 4A). Similarly, the secretion of IgG1 and IgG2 from serum was increased in ΔOmp16-infected mice (Fig. 4A). There was no significant difference in IgG1 and IgG2 levels between wild-type strain- and ΔOmp16-infected mice at one, two or four weeks post-infection (Fig. 4A). These results demonstrate that the level of humoral immunity in ΔOmp16-infected mice was similar to that in mice infected with the parental strain. Moreover, secretion of IFN-γ from serum was elevated to similar levels in both parental strain- and ΔOmp16-infected mice after one and two weeks compared to uninfected mice (Fig. 5A). The expression of IFN-γ mRNA in the spleen was consistent with the IFN-γ levels that were detected in serum (Fig. 5B). In addition, the expression of TNF-α mRNA was increased in parental strain -and ΔOmp16-infected spleens one and two weeks post-infection compared to uninfected mice (Fig. 5C), although there was no significant difference between the two infected samples (Fig. 5C). Finally, we observed that IL-4 expression did not change significantly in spleens infected with either the parental or deletion strain compared to control spleens. Overall, the results indicate that Omp16 did not affect IFN-γ, TNF-α and IL-4 secretion during infection by *B. suis*.

## Discussion

Brucellosis, caused by *Brucella* spp., is a worldwide zoonotic and contagious disease that causes abortion and infertility in animals and chronic debilitating disease in humans that results in serious morbidity, worldwide economic loss, and poverty [7-9]. The available live attenuated vaccines still have some disadvantages in animals, such as serodiagnostic interference and residual pathogenicity [11-13]. In contrast, no patient-friendly therapeutic methods or approved vaccines are reported for humans [9]. Omps, including Omp10, Omp19, Omp25 and Omp31, are involved in outer membrane integrity, virulence and evasion of the host immune system in *Brucella* [24, 25, 28, 33, 34]. Exploration of the function of Omps has been key to understand the pathogenic mechanisms of *Brucella* and the ability of this bacterium to evade the immune system. Omp16, a homolog of peptidoglycan-associated lipoproteins (Pals), is a key factor for *Brucella* survival [28]. A *Brucella* strain deleted of the gene for Omp16 has not been generated in any species. In a previous study, we acquired the ΔOmp16 strain in which *omp16* expression is controlled [29]. Here, we utilized this strain to explore the effect of Omp16 on bacterial virulence and the *Brucella*-mediated immune response in mice. Our results showed that Omp16 is required for *Brucella* survival in the spleen and that the humoral and cellular immune responses mediated by ΔOmp16 are similar to those of the wild-type strain.

The mouse is an important model for persistent *Brucella* infection [7]. Splenomegaly, spleen weight, and survival of bacteria in the spleen are key indicators to assess *Brucella virulence*. Several *Brucella* Omps have been confirmed to be involved in bacterial virulence. For example, the intracellular survival of an Omp10 and Omp19 deletion strain is decreased in the spleen, indicating that this strain is attenuated in vivo [23, 24, 35]. Moreover, Omp25 is an important virulence factor in *Brucella* and a strain deleted of *omp25* is attenuated both in vitro and in vivo [36]. Furthermore, Omp31 plays an important role in *Brucella virulence* [37, 38]. However, several other Omps in *Brucella*, including SP41 and BepC, are not involved in virulence [28]. In a previous study, we found that *Brucella* cells lacking Omp16 presented defects in growth, outer membrane integrity and intracellular survival [29]. Further studies are required to explore the virulence of ΔOmp16 in mice. Using the BALB/c mouse model, we showed that splenomegaly and spleen weight were reduced in ΔOmp16-infected mice and that the bacteria in these mice were cleared quickly compared to parental strain-infected mice. These results are similar to the characteristics of other Omp deletion strains described above. As another key indicator, spleens from ΔOmp16-infected mice exhibited reduced pathological characteristics with a modest increase in the white-to-red pulp ratio and slight white pulp expansion one week post-infection compared to spleens of mice infected with the parental strain. These observations indicate that Omp16 is required to maintain *B. suis* virulence in mice and that ΔOmp16 is attenuated in mice compared to the wild-type strain. The *Brucella* outer membrane is resistant to bactericidal cationic peptide and bactericidal polycations like polymyxin B [39]. *Brucella* LPS, Omp31 and Omp16 mutant strain were more susceptible to polymyxin B in vitro [29, 37, 40]. In addition, intracellular bacteria have developed the capability to adapt to intracellular environments to survive in host cells, including oxidative burst [26]. The survival rate of *Brucella* Omp16 mutant was decreased under oxidative stress [29]. The reduced virulence of the ΔOmp16 in a mouse model of infection may be due to changes in the biological characteristics of bacteria. These findings corroborate our previous results that the intracellular survival rate of ΔOmp16 is decreased compared to wild-type in RAW264.7 macrophages [29].

The humoral response is required to resist intracellular pathogenic bacteria and contributes to the control of bacterial infection [41, 42]. The secretion of IgG2a antibody is associated with cytokines secreted by Th1-type cells, whereas the production of IgG1 antibody is associated with cytokine secretion by Th2-type cells [43]. Wild-type strain and ΔOmp16 induced higher production of IgG1 and IgG2a in infected vs. uninfected mice. However, the production of IgG1 and IgG2a was not significantly different in ΔOmp16-infected mice compared to wild-type strain-infected mice which indicates that Omp16 is not implicated in the humoral response to *Brucella* infection. During *Brucella* infection in the murine model, the host immune response resembles the Th1 type with the secretion of IFN-γ and TNF-α by infected macrophages [44, 45]. As a key cytokine, IFN-γ plays an important role in resisting intracellular pathogenic bacteria, including *Brucella* [46]. IFN-γ^-/-^ mice are unable to control infections, resulting in the death of infected mice at six weeks post-infection [47]. Unlipidated Omp16 (obtained from the bacterial cytoplasm) is able to induce higher protection levels in vivo than lipidated Omp16 (obtained from bacterial membranes), and unlipidated Omp16 mediates IFN-γ production to eradicate *Brucella* [27]. However, we did not observe that the secretion of IFN-γ was significantly different in ΔOmp16-infected mice compared to mice infected with parental strain. This may be due to poor induction of IFN-γ secretion by lipidated Omp16. Furthermore, TNF-α contributes to the control of intracellular pathogenic bacteria. However, neither the secretion of TNF-α nor the production of IL-4 was different in ΔOmp16-infected mice compared to wild-type strain-infected mice. Overall, the results show that the levels of humoral and cellular immunity in wild-type strain- and ΔOmp16-infected mice were similar.

In conclusion, the current study reveals that murine splenomegaly, bacterial load, and histopathological changes in spleen were significantly decreased in a strain deleted of the gene for Omp16 compared to wild-type strain. In addition, the levels of humoral and cellular immunity were indistinguishable in wild-type strain- and ΔOmp16-infected mice. Overall, these findings reveal that Omp16 is required for *Brucella* survival in mice, but that Omp16 is not involved in *Brucella*-induced humoral and cellular immunity. Future studies will focus further on the key role of Omp16 in infection by *B. suis*.

## Figures and Tables

**Table 1:** A list of all primers used in qRT-PCR.

Gene	Forward primer (5’-3’)	Reverse primer (5’-3’)	Length (bp)
IFN-γ	AGCAACAACATAAGCGTCA	GTGGACCACTCGGATGAG	156
TNF-α	TCTCATTCCTGCTTGTGGC	CACTTGGTGGTTTGCTACG	197
IL-4	GTGCAGCTTATCGATGAATCC	AGCCATATCCACGGATGCGAC	287
